# Blood cultures for the diagnosis of multidrug-resistant and extensively drug-resistant tuberculosis among HIV-infected patients from rural South Africa: a cross-sectional study

**DOI:** 10.1186/1471-2334-10-344

**Published:** 2010-12-06

**Authors:** Scott K Heysell, Tania A Thomas, Neel R Gandhi, Anthony P Moll, François J Eksteen, Yacoob Coovadia, Lynette Roux, Palav Babaria, Umesh Lalloo, Gerald Friedland, Sarita Shah

**Affiliations:** 1 Tugela Ferry Care and Research Collaboration (TF CARES), Tugela Ferry, South Africa; 2 University of Virginia, Charlottesville, VA, USA; 3 Albert Einstein College of Medicine and Montefiore Medical Center, New York, NY, USA; 4 Philanjalo Care Center and Church of Scotland Hospital, Tugela Ferry, South Africa; 5 National Health Laboratory Services, Durban, South Africa; 6 Nelson R. Mandela School of Medicine, University of KwaZulu-Natal, Durban, South Africa; 7 Yale University School of Medicine, New Haven, CT, USA

## Abstract

**Background:**

The yield of mycobacterial blood cultures for multidrug-resistant (MDR) and extensively drug-resistant tuberculosis (XDR-TB) among drug-resistant TB suspects has not been described.

**Methods:**

We performed a retrospective, cross-sectional analysis to determine the yield of mycobacterial blood cultures for MDR-TB and XDR-TB among patients suspected of drug-resistant TB from rural South Africa. Secondary outcomes included risk factors of *Mycobacterium tuberculosis *bacteremia and the additive yield of mycobacterial blood cultures compared to sputum culture.

**Results:**

From 9/1/2006 to 12/31/2008, 130 patients suspected of drug-resistant TB were evaluated with mycobacterial blood culture. Each patient had a single mycobacterial blood culture with 41 (32%) positive for *M. tuberculosis*, of which 20 (49%) were XDR-TB and 8 (20%) were MDR-TB. One hundred fourteen (88%) patients were known to be HIV-infected. Patients on antiretroviral therapy were significantly less likely to have a positive blood culture for *M. tuberculosis *(p = 0.002). The diagnosis of MDR or XDR-TB was made by blood culture alone in 12 patients.

**Conclusions:**

Mycobacterial blood cultures provided an additive yield for diagnosis of drug-resistant TB in patients with HIV from rural South Africa. The use of mycobacterial blood cultures should be considered in all patients suspected of drug-resistant TB in similar settings.

## Background

Tuberculosis (TB) is the leading cause of mortality among people living with HIV worldwide [1,2]. Drug-resistant TB has emerged as an important global threat to public health. Although previously considered uncommon in high HIV prevalence settings, there has been a 3-4 fold increase in multidrug-resistant (MDR)-TB prevalence in southern Africa over the past decade [3,4]. In addition, extensively drug-resistant (XDR)-TB has been reported from all countries in southern Africa. MDR and XDR-TB are associated with a much higher mortality than drug-susceptible TB [5], especially among HIV co-infected persons [6].

Prompt diagnosis and treatment are essential to improve drug-resistant TB outcomes, but TB diagnosis in patients with HIV co-infection is challenging, particularly in resource-limited settings [6]. HIV-infected TB patients have higher rates of extrapulmonary disease, atypical clinical presentations, and normal chest radiographs [7-10]. With the emergence of MDR and XDR-TB in HIV-infected populations worldwide [3], it is therefore likely that there will be a consequent rise in extrapulmonary MDR and XDR-TB disease [11]. The diagnosis of drug-resistant TB requires isolation of an organism, or DNA in the case of molecular tests, thus vigorous efforts to obtain a specimen that may yield *Mycobacterium tuberculosis *are needed in settings with a high prevalence of drug resistance.

Mycobacteremia with drug-susceptible TB was described in reports from HIV-infected patients early in the epidemic of HIV from the United States [12,13]. Yet the yield of blood cultures in detecting *M. tuberculosis *can vary between 2%-64% depending on the population of study and suspicion for extrapulmonary TB [14-19]. In addition, HIV-infected patients may be predisposed to other bacterial and fungal bloodstream infections that clinically mimic TB, leading to delays in diagnosis or over-treatment of TB. In sub-Saharan Africa, *M. tuberculosis *bacteremia has been documented in patients with blood stream infections from a referral hospital in Tanzania, in patients with cough from Botswana, and as a critical etiology of sepsis among HIV-infected patients from Uganda, but drug-susceptibility testing (DST) was not performed [20-22]. To date, no study has reported the yield of blood cultures for the detection of MDR and XDR-TB.

Over 650 patients with MDR and XDR-TB have been identified from the rural Tugela Ferry area of KwaZulu-Natal, South Africa, where HIV co-infection rates exceed 90% [5,23]. Mycobacterial blood cultures have been used routinely in Tugela Ferry since 2006 in an attempt to improve case detection of drug-resistant TB. We sought to quantify the yield of blood cultures for MDR and XDR-TB, compare the yield to that of sputum culture, and identify risk factors for *M. tuberculosis *bacteremia in this population in order to guide clinical practice and public health policy.

## Methods

### Setting

The Church of Scotland Hospital (COSH) in Tugela Ferry is a 355-bed facility serving a population of 200,000 Zulu people. The local incidence of TB is estimated at 1,100 per 100,000 population, with approximately 80% of TB cases being HIV coinfected[24]. Onsite diagnostics include smear microscopy for acid-fact bacilli of sputum and cerebrospinal fluid specimens. Sputum and other non-sputum fluid specimens requiring culture and DST are sent to the provincial TB referral laboratory in Durban (approximately 180 kilometers away) on a daily basis. Since June 2005, all TB suspects presenting to COSH are requested to give two 'spot' sputum specimens, one for onsite smear microscopy, and one for mycobacterial culture and DST.

### Study design

We performed a retrospective, cross-sectional study of all drug-resistant TB suspects in whom at least one mycobacterial blood culture was sent from September 1^st^, 2006 to December 31^st^, 2008. Clinicians defined a drug-resistant TB suspect based on the presence of TB symptoms (e.g., cough, nightsweats, weight loss) with one or more of the following additional criteria: advanced HIV/AIDS, a prior history of TB treatment, or persistent symptoms despite one month or more of drug-susceptible TB treatment. If a sputum culture was available it was included for analysis if collected within two weeks before or after the date of collection of the blood culture.

Medical chart review was performed to obtain demographic, clinical and microbiological information. Specific data extracted included age, gender, HIV status, receipt and duration of antiretroviral treatment, CD4 cell count (cells/mm^3^) prior to blood culture collection, history of TB treatment, TB treatment status at time of blood culture collection, and physician's comments of signs of extrapulmonary TB.

### Definitions and outcome measures

Patients were categorized as being extrapulmonary TB suspects if a physician had documented specific extrapulmonary organ involvement that was suggestive of TB (e.g., pericardial effusion or abdominal lymphadenopathy on ultrasound) or if the physician documented a suspicion for extrapulmonary TB in the chart. MDR-TB was defined as resistance to at least isoniazid and rifampicin, while XDR-TB was defined as resistance to at least isoniazid, rifampicin, kanamycin and ofloxacin [25]. Susceptibility testing to other second-line TB drugs was not routinely done.

The primary outcome was the yield of blood cultures for drug-resistant TB, defined as the proportion of blood cultures that were positive for MDR or XDR-TB. Secondary outcomes included: 1) risk factors of *M. tuberculosis *bacteremia, and 2) comparison of blood culture to sputum culture for additive yield of blood in detection of *M. tuberculosis *and drug resistance.

### Laboratory methods

All patients had 5 ml of blood collected and inoculated into mycobacterial blood culture bottles (BACTEC MycoF-lytic) and placed in a darkened storage container at room temperature prior to transport to the provincial referral laboratory. Blood culture bottles were cultured using the automated BACTEC 9240 system in which specimens are continuously monitored for growth up to 42 days [26]. All positive cultures were confirmed by niacin accumulation and nitrate reductase methods. DST was performed on all specimens positive for *M. tuberculosis *using the 1% proportional method [27] on Middlebrook 7H11 agar to: isoniazid (critical concentration, 0.2 μg/ml), rifampicin (1 μg/ml), ethambutol (7.5 μg/ml), ofloxacin (2 μg/ml), kanamycin (6 μg/ml) and streptomycin (2 μg/ml). Non-tuberculous mycobacteria (NTM) were not further speciated.

Sputum samples were refrigerated before and during transport to the provincial TB referral laboratory. Upon receipt, the specimen was digested and decontaminated with the N-acetyl-L-cysteine-sodium hydroxide method and smears were prepared for auramine staining. The remainder of the deposit was transferred for liquid culture in the automated BACTEC MGIT 960 system. DST was completed on all positive specimens after secondary inoculation on to Middlebrook 7H11 agar and using the 1% proportional method for the drugs as described with blood specimens.

### Statistical analysis

Yield of *M. tuberculosis *detected by blood and sputum was calculated using simple frequencies and proportions. Demographic and clinical characteristics were compared with the chi-square statistic or the Mann-Whitney U test for non-parametric data. Bivariate and multivariate logistic regression were employed to determine risk factors for *M. tuberculosis *bacteremia. All tests for significance were 2-sided with a p-value < 0.05 considered significant. For variables with >10% missing data, tests of interaction were performed when appropriate. The multivariate model included any variable with p-value < 0.1 in bivariate analysis and any pertinent clinical and demographic characteristics. All analysis was performed using SPSS Statistics 17.0 software.

### Ethical considerations

The study was approved by the biomedical research ethics committees of the University of KwaZulu-Natal, Albert Einstein College of Medicine, and Yale University.

## Results

One-hundred thirty patients suspected of drug-resistant TB had mycobacterial blood cultures performed during the study period and were included for analysis. All patients had only one blood culture specimen. There were 73 males (56%); the median age was 31.5 years (Interquartile range [IQR] 27-38) and 8 (6%) patients were less than 12 years of age (Table 1). HIV-infection was confirmed in 114 (88%) patients. The CD4 cell count was available in 63 (55%) HIV-infected patients, with a median cell count of 100 cells/mm^3 ^(IQR 28-190). Fifty-three (46%) HIV-infected patients were on antiretroviral therapy at the time of blood culture collection. The median duration of antiretroviral therapy for patients on treatment was 15.4 weeks (IQR 4.7-31.0).

**Table 1:** Demographic and clinical characteristics compared by patients with and without M. tuberculosis (MTB) bacteremia

Characteristic	Total n (%)	Blood culture positive for MTB, n (%)	Blood culture negative for MTB, n (%)	Bivariate relative risk [95% confidence interval], p-value
Total	n = 130	n = 41	n = 89	
Sex:				
Male	73 (56)	22 (54)	51 (57)	1.16 [0.55-2.4] p = 0.70
Female	57 (44)	19 (46)	38 (43)	referent
Age range (years):				
0-14	8 (6)	1 (2)	7 (8)	0.42 [0.05-3.7] p = 0.43
15-25	15 (11)	7 (17)	8 (9)	2.57 [0.81-8.2] p = 0.11
26-35	67 (52)	17 (41)	50 (56)	referent
36-45	28 (22)	11 (27)	17 (19)	1.9 [0.75-4.9] p = 0.18
> 45	12 (9)	5 (12)	7 (8)	2.1 [0.59-7.5] p = 0.25
Prior history of TB treatment				
Yes	42 (32)	15 (37)	27 (30)	1.33 [0.61-2.9] p = 0.48
No	88 (68)	26 (63)	62 (70)	referent
Currently on first-line TB treatment				
Yes	89 (69)	28 (68)	61 (69)	0.99 [0.45-2.2] p = 0.98
No	41 (31)	13 (32)	28 (31)	referent
Duration of TB treatment for patients on treatment (median weeks) [IQR]	8 [4.0-20.0]	10 [4.0-24]	8 [5.5-13.5]	p = 0.76
Clinical features extrapulmonary TB				
Yes	45 (35)	19 (46)	26 (29)	2.10 [0.97-4.5] p = 0.06
No	85 (65)	22 (54)	63 (71)	referent
HIV status:				
Positive	114 (88)	34 (83)	80 (90)	p > 0.99
Negative	1 (1)	0 (0)	1 (1)	referent
Unknown	15 (11)	7 (17)	8 (9)	p > 0.99
CD4 count^a ^(median cells/mm^3^) [IQR]	100^b ^[28-190]	92 [81-174]	104 [21-222]	p = 0.82
CD4 count cells/mm^3^				
0-100	32 (51)	24 (50)	8 (54)	1.92 [0.35-10.5] p = 0.45
101-200	17 (27)	12 (25)	5 (33)	2.73 [0.44-17.1] p = 0.29
> 200	14 (22)	12 (25)	2 (13)	referent
Receiving antiretroviral therapy^c^				
Yes	53 (47)	9 (26)	44 (55)	0.29 [0.12-0.71] p = 0.005
No	61 (53)	25 (72)	36 (45)	referent
Duration of antiretroviral therapy(median weeks) [IQR]	15 [4-31]	16 [7-32]	15 [5-30]	p = 0.92

IQR = interquartile range.

^a ^Available in 63 (55%) of 114 HIV-infected patients.

^b ^CD4% in 3 pediatric cases (7.0%, 6.0% and 7.9%)

^c^Available in 114 (100%) HIV-infected patients.

Of the 130 patients, 88 (68%) had no prior history of TB, although 89 (69%) patients were failing drug-susceptible TB treatment at the time of blood culture collection. The median duration of TB treatment for these patients was 8.0 weeks (IQR 4.0-20.0). Forty-five (35%) patients were suspected to have extrapulmonary TB.

### Overall yield of mycobacterial blood cultures

Of 130 blood culture specimens, *M. tuberculosis *was isolated in 41 (32%) (Table 2). Of the 41 blood cultures that yielded *M. tuberculosis*, 28 (68%) were drug-resistant TB. Specifically, 20 (49%) specimens were XDR-TB and 8 (20%) were MDR-TB (Table 2). Among the blood cultures positive for XDR-TB, 18 (90%) of 20 were resistant to all six drugs tested.

**Table 2:** Overall yield of mycobacterial blood cultures for diagnosis of drug-susceptible, multidrug-resistant (MDR) and extensively drug-resistant (XDR) tuberculosis (TB)

	n (%)
Blood culture result (n = 130):	
*M. tuberculosis*	41 (32)
Non-tuberculous mycobacteria	3 (2)
*Cryptococcus *sp.	3 (2)
Other^a^	5 (4)
Negative	78 (60)
Drug-susceptibility results among *M. tuberculosis *isolates (n = 41):	
XDR-TB	20 (49)
MDR-TB^b^	8 (20)
Drug-susceptible	10 (24)
Incomplete^c^	3 (7)

^a ^Other bacterial species, possible contaminants.

^b ^All MDR-TB specimens were also resistant to streptomycin.

^c ^*M. tuberculosis *identified, susceptibility results not available.

Of the remaining positive blood cultures, NTM were found in 3 (6%) specimens and *Cryptococcus *species was found in 3 (6%) specimens. In the six study patients with *Cryptoccocus *species or NTM detected in the blood, five were currently receiving first-line TB therapy at the time of blood culture collection yet none of the patients had culture documentation of *M. tuberculosis*.

### Risk factors for M. tuberculosis bacteremia

There were significantly more patients with *M. tuberculosis *bacteremia who were extrapulmonary TB suspects, OR 2.1 [0.97-4.5; p = 0.06]; adjusted OR 2.3 [1.0-5.4; p = 0.05]. Among HIV-infected patients, those on antiretroviral therapy for any duration at the time of blood culture collection were significantly less likely to have *M. tuberculosis *bacteremia, OR 0.29 [95% CI, 0.12-0.71; p = 0.005]; adjusted OR 0.22 [0.08-0.58; p = 0.002] (Table 1). Though age was not a significant risk factor, it was found that *M. tuberculosis *was cultured from the blood in patients as young as 8 years (MDR-TB) and as old as 62 years (XDR-TB).

### Comparison of blood to sputum cultures for MDR-TB and XDR-TB yield

Of the 41 patients with *M. tuberculosis *bacteremia, there were 23 patients that also had a sputum sample collected for comparison (Table 3). With two patients, the sputum sample was negative but the blood cultures revealed XDR-TB in one and MDR-TB in the other. DST was not completed in one patient's sputum sample but the blood culture revealed XDR-TB. Among all patients in whom DST was completed in both the blood and sputum sample, the DST results were identical. Considering the 21 patients where sputum cultures were either negative, DST was incomplete, or sputum was not collected, there were 9 (43%) blood cultures that diagnosed drug-susceptible TB and 12 (57%) that diagnosed MDR or XDR-TB (Table 3). Despite extrapulmonary TB suspects being at higher risk of *M. tuberculosis *bacteremia, in patients with both a blood and a sputum culture positive for *M. tuberculosis*, only 24% were suspected of extrapulmonary TB.

**Table 3:** Sputum culture results among patients with a positive blood culture for M. tuberculosis (n = 41)

		Blood culture drug-susceptibility test result			
		DS TB	MDR-TB	XDR-TB	DST incomplete
Sputum culture result	DS TB	4			
	MDR-TB		4		
	XDR-TB			12	
	DST incomplete^a^			1	
	No growth		1	1	
	Not collected	6	3	6	3

Additive yield of blood by DST category		6 (60%)	4 (50%)	8 (40%)	3 (100%)

DS TB = drug-susceptible TB.

MDR-TB: Multidrug-resistant tuberculosis.

XDR-TB: Extensively drug-resistant tuberculosis.

DST: drug-susceptibility test. ^a ^*M. tuberculosis *identified, but DST not available.

## Discussion

We found that among a predominantly HIV-infected population of patients suspected of drug-resistant TB, MDR-TB and XDR-TB were isolated in nearly 70% of all positive *M. tuberculosis *blood cultures. Importantly, among patients with MDR or XDR-TB bacteremia, in over half of those in whom a sputum culture was unavailable, the blood culture was the only means of drug-resistant TB diagnosis. Bacteremia with XDR-TB was more common than MDR-TB, but reflective of community trends from sputum diagnosis in Tugela Ferry [28].

Current guidelines suggest the use of mycobacterial blood cultures may be beneficial in suspected extrapulmonary TB, but do not address the use in all HIV-infected persons or those suspected of drug-resistant TB [29]. A recent comprehensive screening study of HIV-infected ambulatory persons from Southeast Asia found only a 5% incremental yield of blood cultures for TB diagnosis among those with two negative sputum smears; DST results were not provided [19]. In contrast, the results from our study population are likely reflective of advanced immunosuppression, prolonged TB illness prior to blood culture collection, and the high pretest suspicion of drug-resistant TB. The additive yield of blood cultures is likely to vary in other regions with differing disease epidemiology. Nonetheless, these results suggest that *M. tuberculosis *bacteremia is likely to be present in drug-resistant TB suspects at higher rates than clinically suspected. Thus, we feel that these results are generalizable to other populations in sub-Saharan Africa where TB/HIV co-infection rates are high and the incidence of drug-resistant TB may be increasing.

The bulk of the additive yield for MDR and XDR-TB in blood compared to sputum cultures was found in patients that did not have a sputum sample collected. The most common reason to not have a sputum sample collected in this hospital is the patient's inability to expectorate due to an absence of cough or marked physical disability; however, due to the retrospective nature of this study, we cannot confirm the reasoning for an individual patient. Standard of practice in other settings is to collect two or more sputum samples for microscopy and/or TB culture as a means of increasing yield. Further prospective study is warranted to determine how multiple sputum samples would affect the comparative yield to blood culture in similar populations with advanced HIV. Blood is an easily accessible fluid and carries the additional advantage of not requiring cold storage for transport. Additionally, the cost of analyzing a mycobacterial blood culture with the National Health Services Laboratory in South Africa is no more expensive than MGIT analysis of a sputum specimen.

In this study, the majority of patients with *M. tuberculosis *bacteremia were not suspected to have extrapulmonary TB. Mycobacterial culture of lymph node aspirates and pleural fluid were available to clinicians during this study period [11], yet in only three patients was an aspirate performed and all were concordant with blood culture results. Indeed, the minority of patients with positive blood cultures and sputum cultures for *M. tuberculosis *were suspected of extrapulmonary TB. Thus, our findings suggest that many patients with pulmonary TB in this setting may also harbor unrecognized *M. tuberculosis *bacteremia. Detection of otherwise occult *M. tuberculosis *bacteremia regardless of DST, in a patient without suspected extrapulmonary TB may prompt a more exhaustive search for an extrapulmonary focus which could alter treatment and carry important implications for monitoring and clinical outcome.

Patients on antiretroviral medication at the time of blood culture collection were significantly less likely to have *M. tuberculosis *bacteremia. Earlier studies of *M. tuberculosis *bacteremia in similar populations in Africa were carried out prior to widespread availability of antiretrovirals and therefore this association could not have been documented until now [20,21]. Our findings lend further support to the growing body of evidence for early initiation of antiretrovirals in the treatment of TB and HIV co-infected patients [30]. Notably, the median duration of antiretroviral use in our study population was 15 weeks, a reasonable timeframe to present with immune reconstitution inflammatory syndrome (IRIS). We suspect that some patients on antiretroviral therapy who were culture-negative for TB may have actually presented with IRIS, a condition which may share signs and symptoms with TB and drug-resistant TB; however complete follow-up data were not available for confirmation. Interestingly, there was no difference in CD4 count among patients with and without *M. tuberculosis *bacteremia. One explanation is, in accord with national guidelines, the CD4 count is only checked twice annually; thus the CD4 count recorded may be falsely low for those patients that initiated antiretroviral therapy within the prior six months. Alternatively, in some patients early restoration of lymphocyte function may precede restoration of total lymphocyte count.

One of the primary limitations of the study, given its retrospective design, was that the decision of blood culture collection was dependent upon the attending clinician and therefore additional patients suspected of drug-resistant TB may not have had blood cultures sent and were not included in the study. Additional factors that influenced the decision to pursue the investigation for *M. tuberculosis *bacteremia may not have been captured. It is also a possibility that blood cultures may have been preferentially pursued in patients in whom a diagnosis was not as readily made by sputum analysis. Therefore, only prospective study of simultaneous blood and rigorous collection of multiple sputum samples in all drug-resistant TB suspects would allow determination of a true incremental yield in this setting.

## Conclusions

In summary, mycobacterial blood cultures diagnosed MDR and XDR-TB in a substantial number of patients predominately HIV-infected and suspected of drug-resistant TB from rural South Africa. Bacteremia with drug-susceptible and drug-resistant TB was not restricted to patients suspected of extrapulmonary TB, as many patients with sputum culture confirmed pulmonary TB also had *M. tuberculosis *bacteremia. The adjunctive use of mycobacterial blood cultures should be considered in all patients suspected of drug-resistant TB, particularly in those unable to expectorate. In many regions of Africa and the developing world, culture and DST are not routinely performed for the diagnosis of TB despite the inferior sensitivity of routine sputum microscopy and the inability of microscopy to detect drug-resistant TB. Expanded access to culture and DST of sputum in South Africa has been projected to save 47,955 lives and avert 7,721 new MDR-TB cases over the next 10 years [31]. Our finding that a significant proportion of drug-resistant TB suspects had MDR-TB or XDR-TB bacteremia underscores the need for more widespread use of culture and DST for both sputum and blood specimens.

## Competing interests

The authors declare that they have no competing interests.

## Authors' contributions

SKH conceived of the study design, performed data analysis and drafted the manuscript. TAT, NRG and SS participated in study design, data analysis and critical revision of the manuscript. FJE, APM and PB participated in study design, acquired patient related data, and provided critical revision of the manuscript. YC and LR performed specimen processing, culture and drug-susceptibility testing. UL and GF participated in study design and critical revision of the manuscript. All authors read and approved the final manuscript.

## Pre-publication history

The pre-publication history for this paper can be accessed here:

http://www.biomedcentral.com/1471-2334/10/344/prepub
